# Self-Control, Intoxication and Violence: A Cross-National Comparison of English and Swedish Adolescents

**DOI:** 10.1007/s43576-026-00207-6

**Published:** 2026-02-24

**Authors:** Kyle Treiber, Robert Svensson

**Affiliations:** 1 Institute of Criminology, University of Cambridge, Cambridge, UK; 2 Department of Criminology, Malmö University, Malmö, Sweden

**Keywords:** PADS +, MINDS, Situational action theory, Self-control, Alcohol, Intoxication, Violence, Adolescents

## Abstract

This paper draws upon situational action theory (SAT) to frame and test the relationship between alcohol intoxication and violence in adolescents from on-going longitudinal studies in two European countries: the Malmö Individual and Neighbourhood Development Study (MINDS, N = 514) based in Malmö, Sweden, and the Peterborough Adolescent and Young Adult Development Study (PADS + , N = 704) based in Peterborough, UK. Using data from participants at ages 15–16, this paper assesses if SAT’s explanation for the relationship between adolescents’ alcohol use and violence is applicable in both countries, despite significant differences in their rates of alcohol use and violence. Theorizing that alcohol use influences violence by (temporarily) reducing a person’s ability to exercise self-control, the paper anticipates and finds a robust interaction between alcohol use and self-control in predicting violence in both samples. To account for differences in the samples’ need to exercise self-control, the paper also examines differences in violence-relevant moral attitudes and exposure. By contextualizing intoxication, this analysis is able to account for all statistical differences in violence between British and Swedish adolescents, suggesting that while British and Swedish youths differ in their *need* to exercise self-control to choose non-violent alternatives, there are no differences by country in the relationship between adolescents’ ability to exercise self-control, frequency of intoxication, and choice of violence. This is consistent with SAT’s proposition that the process through which people come to see and choose their action alternatives is universal, while the inputs to that process may vary by population and context.

## Introduction

Alcohol consumption and violence are frequently cited among the most significant and costly social problems for adolescents (Institute of Alcohol Studies, 2024; WHO, 2024a). Many large international surveys and cross-national comparisons find that British adolescents top the charts for rates of problematic alcohol use and violence (e.g., Bräker et al., 2015; Hibell et al., 2012; Steketee, 2012; Wikström & Svensson, 2008), though both have declined since the turn of the century (e.g., Humphreys et al., 2019; Ng Fat et al., 2018; Oldham et al., 2020). By contrast, Swedish youths generally report lower than average levels of intoxication and violence (e.g., Enzmann et al., 2010; Hemström, 2002; Junger-Tas, 2012; Leifman, 2002; Ring & Andersson, 2010), and particularly significantly declining trends in both (Carlson, 2019; e.g., Loy et al., 2021; Pape et al., 2018). A robust body of literature highlights the link between intoxication and violence (e.g., Bergmark et al., 2005; Bye & Rossow, 2010; Felson et al., 2011; Wolf et al., 2014). Can the causal process through which violence comes about be identified, and the role which intoxication plays? Is it the same in both Sweden and the UK?

This paper has three main aims that may enhance conceptualization of the relationship between adolescents’ alcohol use and levels of violence cross-nationally:

1. To explore the general relationship between frequency of intoxication, ability to exercise self-control, and violence
2. To analyse cross-cultural differences between British and Swedish youths’ drinking habits, levels of self-control, and violence
3. To take into account situational factors^1^ that may determine the relevance of self-control and intoxication for violence in different contexts

### The Macro-Level: Patterns of Violence and Alcohol Use

#### Violence

International studies consistently find that adolescents from Anglo-American countries, including the UK, self-report higher levels of violence than young people from most other countries, including Sweden (UNICEF, 2007). A direct comparison found British adolescents (from the Peterborough Youth Study, 2000) were more than 2.5 times as likely as Swedish adolescents (from the Eskilstuna School Study, 1996/1997) to have hit someone in the past year (24.0% vs. 9.9%) (Wikström & Svensson, 2008). Large differences are also observable in victimisation rates, with Brits reporting unusually high rates, and Swedes unusually low^2^ (e.g., Gruszczyńska et al., 2012).

#### Alcohol use

Europe has been cited as having the highest per capita levels of alcohol consumption in the world (Järvinen & Room, 2007; WHO, 2005, 2011, 2018, 2024b), and Brits as being some of Europe’s heaviest consumers (BMA, 2008); Swedes, on the other hand, are some of the lightest (Leifman, 2002). Youths in the UK are more likely to be drinkers than youths in Sweden, although youths in both countries report easy access to alcohol despite similar age restrictions (Bräker et al., 2015; Hibell et al., 2012; Steketee, 2012). People also drink more frequently in the UK than Sweden (Currie et al., 2012; Knibbe et al., 2006; Mäkelä et al., 2005); 1 in 3 British but only 1 in 10 Swedish youths drink weekly (Hibell et al., 2012; NHS, 2011, 2024; Ring & Andersson, 2010). The European Comparative Alcohol Study (ECAS) found Brits reported the highest and Swedes the lowest rates of regular alcohol consumption across 15 countries (Hemström, 2002). However, both tend to drink heavily per occasion (Bye & Rossow, 2010; Hemström, 2002; Knibbe et al., 2006), which may have particular relevance for violence, as binge drinkers are more likely to commit violence than non-binge drinkers (Matthews & Richardson, 2005).

#### Alcohol and Violence

National levels of violence are related to national levels of alcohol consumption (MacAndrew & Edgerton, 2003; Room & Mäkelä, 2000; van Dijk et al., 2007), most strongly in countries where consumption is characterized by more binge drinking (Bergmark et al., 2005; Bobak, 2004; Bye & Rossow, 2010; Felson et al., 2011; WHO, 2005). In a study of six European countries, British adults reported fighting as a consequence of alcohol more often than adults from any other country (7.5%, M = 3.6%) and more than five times as often as adults from Sweden (1.3%) (Hemström, 2002).

Some models argue that problematic alcohol use and problematic behaviour, such as violence, are related because they are outcomes of the same driver/s (e.g., low self-control; Gottfredson and Hirschi 1990). Yet the considerable variation observed in the relationship between alcohol use and violence indicates this relationship is more complex; many people who exhibit problematic alcohol use do not exhibit violence, and many people who exhibit violence do not exhibit problematic alcohol use (e.g., Bukten et al., 2024; Fazel et al., 2017; Newbury-Birch et al., 2022). Much is also known regarding the physiological effects of alcohol consumption and their relevance for behaviour, including violence, and yet ‘most drinking occasions do not result in aggression or violence, and drinking is neither necessary nor sufficient to cause violence’ (Plant et al., 2002, p. 207; see also Graham et al., 1996; Hoaken et al., 1998). Taken together, the picture is complex and warrants deeper analytical consideration.

Better understanding how alcohol consumption can lead to violence may help us better explain patterns of violence and alcohol consumption observed within and between countries. We propose that situational action theory can help us better capture the nuance of the relationship between alcohol and violence by explicitly framing the role of alcohol consumption in the micro-level processes through which people come to see and choose violence as an option for action.

### The Model: Situational Action Theory

There is no reason to think alcohol would lead to violence amongst British youths via different causal pathways than it does amongst Swedish (or any other) youths; at a fundamental level, the action process through which youths interact with their environments and come to see and choose their action alternatives will be the same, as will the general psychophysiological effects of alcohol. What could differ is the content informing the action process, i.e., relevant characteristics of actors and action settings.

Situational action theory (SAT; see Wikström et al., 2024) provides a framework detailing how people come to see and choose their options for action, and the conditions under which this may lead to violence. According to SAT, people commit violence because they see it as an acceptable response, given the circumstances, to a motivation (typically a provocation); or they recognize it as potentially unacceptable, but consider it (e.g., due to predilections or pressures) and there are no sufficient controls to dissuade them. Whether people see violence as an alternative, and choose it over other alternatives, depends on their interaction with the settings and circumstances they encounter. This interaction is shaped by key personal characteristics – their personal morality (what they consider acceptable) and ability to exercise self-control (to act in accordance with their morality when pressured to do otherwise) – and key environmental features – the setting’s moral rules (what is considered acceptable) and ability to deter rule-breaking (to influence setting users to act in accordance with the setting’s moral rules when they are inclined to do otherwise).

We posit that within this framework the relationship between alcohol and violence can be understood primarily through the effects of intoxication on people’s ability to exercise self-control.^3^ According to SAT, self-control is a situational concept – a characteristic of the person–environment interaction (Wikström & Treiber, 2007). People will differ in their need to exercise self-control depending on whether, and how seriously, they consider breaking a personal moral rule in response to an environmental inducement. Whether a person has the *ability* to exercise self-control and choose an alternative consistent with his/her personal morality depends on his/her ability to exercise a *sufficient level* of self-control to meet situational needs.

Importantly, SAT’s concept of self-control is distinct from the concept depicted in Gottfredson and Hirschi’s general theory of crime (GTC; Gottfredson & Hirschi 1990), with important implications for explaining self-control’s role in crime causation (see Wikström & Treiber, 2007; Wikström et al., 2024: 55–64). In SAT, the concept of self-control is distinguished from that of personal morality, with implications for the relevance of self-control for some acts of crime; in GTC personal morality is subsumed within the concept of self-control, which is seen as relevant for all acts of crime (Gottfredson & Hirschi 2020). In SAT, self-control controls people’s adherence to their personal morals when they encounter pressures to contravene them, acknowledging that people differ in their motivations; in GTC, self-control controls the expression of self-interest, which is seen as a universal motivator (Gottfredson & Hirschi 1990; 2020). Thus in SAT, self-control reflects a situational mental process by which people manage conflicting rule-guidance when needed, not an individual trait as it is depicted in GTC, nor limited as in GTC to attention paid to the consequences of action (Wikström et al., 2024: 61–64).

People differ in their *ability to exercise self-control* depending on their *executive capabilities* – higher order cognitive abilities which facilitate goal maintenance, abstract information processing and action guidance. These functions are governed by the prefrontal cortex (PFC), a brain region implicated in the processing and integration of internal and external information, including rules of conduct, and the perception and selection of action alternatives (Treiber, 2011, 2017b; Wikström & Treiber, 2007). The PFC is also strongly and specifically affected by alcohol. Hence it is via these functions that alcohol may undermine the ability to exercise self-control and increase susceptibility to external inducements, e.g., to violence.

### The Micro-Level: Linking Alcohol to Violence via Self-Control

#### Psychopharmacological Effects

Of all recreational drugs, alcohol has the most consistently evidenced relationship with violence (Hoaken & Stewart, 2003). Alcohol both stimulates and sedates the brain, leading to a wide-range of effects on cognition and behaviour (Hoaken et al., 1998; Nutt, 1999; Pihl et al., 2003; Zeigler et al., 2005). Importantly, ‘most people who drink, even heavily, do not become aggressive’ (Pihl et al., 2003, p. 302). In this paper we propose that those who do may be identified, in part, by their poor ability to exercise self-control.

Alcohol’s main psychopharmacological effects increase the likelihood of seeing and choosing violence as an alternative under certain conditions (Pihl et al., 1993). Alcohol stimulates psychomotor activity, increasing sensation-seeking and risk-taking, in part by enhancing reward sensitivity (Hoaken & Stewart, 2003; Pihl et al., 1993), making intoxicated people more reactive to motivational cues and potentially less attentive to inhibitory ones (Giancola & Zeichner, 1997). Alcohol also retards the threat response system, which may impair appraisal of provocative situations (Pihl et al., 2003; Zeichner & Pihl, 1979, 1980). Together these effects exacerbate alcohol’s most significant psychopharmacological effect: its disruption of cognitive capacities regulating motivation and action (executive capabilities).

Alcohol appears to have a particular and even specific effect on PFC and hence executive functioning (Hoaken & Stewart, 2003; Peterson et al., 1990; Pihl et al., 2003), affecting perception (e.g., of threats), evaluation (e.g., of motivational cues) and the selection of ‘suitable response strategies’ (Oscar-Berman & Marinković, 2007; Pihl et al., 2003). This leads to the dysregulation of impulse control and impairs deliberation, undermining a person’s ability to resist engaging in activities he/she may recognize (or would otherwise have recognized) as inappropriate. These effects may be particularly significant for those who have poorer prefrontal/executive functioning generally (but only under conditions in which they are provoked and there is something to control) (e.g., Giancola, 2004; Lau et al., 1995; Pihl et al., 2003).

Alcohol’s psychopharmacological effects may be particularly problematic for adolescents. Developmental changes in the coordination of motivational and cognitive control processes during adolescence means youths already experience naturally heightened reward sensitivity and novelty seeking and less cognitive control, which alcohol can exacerbate, leading to particularly uncontrolled behaviour (Spear, 2018; Treiber, 2017a; Treloar et al., 2017). Adolescents are also less sensitive to alcohol’s sedative effects and can continue functioning in settings where they create/encounter provocation (Spear, 2011). They can also consume more alcohol in a single session, making them vulnerable to acute conditions like asphyxiation and alcohol poisoning (Nutt, 1999) as well as neurotoxic effects, which primarily impact the PFC, and have long-term effects on motivational and action control (Crews et al., 2004; El Marroun et al., 2021; Pérez-García et al., 2022; Zeigler et al., 2005).

#### Beyond Psychopharmacology

The effects of alcohol on action cannot be explained by its physiological effects alone; ‘it is well established that alcohol-related aggression involves drug effects (i.e., effects of intoxication), situational factors, and predisposition of the drinker’ (Graham et al., 1996, p. 522). While experimental research suggests a dose–response effect of alcohol on aggression (Giancola, 2004; Graham et al., 1996), this is only observed for certain people under certain conditions. Key factors which have been identified are consistent with those SAT associates with the exhibition of self-control, e.g., executive capabilities, which determine the *ability* to exercise self-control; and situational provocation, which determines the *need* to exercise self-control (Wikström & Treiber, 2007; Wikström et al., 2024).

People’s alcohol use and behavior when intoxicated depends on the drinking context, (e.g., whether they are on a date, drowning the sorrows of a break-up, or cheering on a sports team); who is present (e.g., friends, work colleagues, rivals); and the surrounding circumstances (e.g., relationship histories, sociohistorical factors, personal preferences). Each of these drinking contexts exhibits a different moral context – different kinds and quantities of frictions (motivational cues) which may be sources of provocation, different moral norms which influence perception of acceptable responses, and different levels of enforcement. Different moral contexts differentially encourage or discourage certain behaviours, including alcohol consumption, violence, and the exhibition of self-control.

Drinking in westernized countries often takes place during evening socializing, with youths more likely to drink alcohol in public spaces (Degenhardt et al., 2015; Storvoll et al., 2010), where the potential for friction and hence provocation increases, especially when people are intoxicated. Outdoor settings may have particularly weak moral contexts (Wikström et al., 2012b), especially in the presence of peers who encourage rule-breaking and absence of supervision and external controls.

Some drinking contexts encourage relaxation of social norms (Felson et al., 2011; Järvinen & Room, 2007; MacAndrew & Edgerton, 2003; Room, 2001). This may broaden the action alternatives people perceive and consider, affecting how much self-control is needed and how effectively it can be exercised. This could extend to the use of violence in response to frictions such as threats or insults; a person may see aggression as less reprehensible when an aggressor is intoxicated, and therefore less need to exercise self-control. People who see increased aggression/violence as a predictable or even normal outcome of intoxication may be more likely to become aggressive/violent when drinking (Graham et al., 1996). Their own violence may be less morally inhibited, or they may perceive the actions of intoxicated others as aggressive. Across more than 30 European countries, two-thirds of 15–16 year-olds reported believing alcohol plays a major role in violence (Felson et al., 2011; Hibell et al., 2012). Clarifying the relationship between alcohol and violence has the potential to adjust these perceptions and defuse criminogenic situations.

Many drinkers may not be prone to breaking rules, but those with drinking problems, personal characteristics including a poor ability to exercise self-control, or who are ‘looking for trouble’ will be disproportionately selected into drinking settings. This selection may be particularly strong among adolescents as underage drinking is an illicit act (Felson et al., 2011), potentially inflating the relationship between alcohol use and violence.

Of all the psychopharmacological and contextual factors that may link alcohol use and violence, provocation appears to have one of the strongest and most consistent effects (Giancola, 2004). Experiments find that intoxication significantly increases aggression only for certain people and *only in the presence of provocation* (Graham et al., 1996). This is consistent with SAT’s emphasis on the importance of exposure and differential motivation; drinking contexts may be characterized by frictions that those who are intoxicated are more likely to find provoking, and moral contexts in which they are more likely to see violence as acceptable. Intoxication would reduce their ability to exercise self-control and resist inducements to violence, and the lower their general ability to exercise self-control, the more susceptible they may be to choosing violence when intoxicated.

Studies of alcohol and violence evidence their complex relationship and underscore the need for a nuanced analysis of their interplay in the action process which accounts for personal, contextual and situational factors.

### Do Brits and Swedes Differ in Their Ability to Exercise Self-Control?

There are few direct cross-national comparisons of Brits’ and Swedes’ ability to exercise self-control, but existing research suggests that young Brits may (generally) be comparatively poor at exhibiting self-control, while young Swedes are comparatively adept. The ISRD-2 reports the lowest levels of self-control in Anglo-Saxon countries (Ireland, Canada and the USA), and the highest levels in Northern Europe (including Sweden) (Marshall & Enzmann, 2012).

### Do Brits and Swedes Differ in the Relationship Between Their Ability to Exercise Self-Control and Violence?

Rebellon et al. (2008) report a stronger link between self-control and violence amongst British university students (one of the strongest amongst the 32 countries they studied) than in Sweden (one of the lowest). SAT suggests the ability to exercise self-control is only relevant for those with weak morality (Wikström, 2010). This has been supported by a number of recent studies^4^ (Li, 2004; Svensson et al., 2010; Wikström & Svensson, 2008; Wikström et al., 2012b) and implies that for populations with generally strong morality, the link between self-control and violence may be attenuated. We account for this by including measures of morality and exposure in our analyses. By delineating the process through which we theorize self-control and violence are related, we can better assess if differences in the strength of their relationship in different contexts is nonetheless explicable through a single process, or if alternative explanations (different causal models) are needed.

## Conclusions

Previous research suggests rates of alcohol use and violence are significantly higher in the UK than in Sweden and, conversely, people’s general ability to exercise self-control is lower. We propose these patterns may be interrelated through the effects of intoxication on self-control, which temporarily increase certain people’s propensity to choose to commit acts of violence. Our theoretical framework highlights the importance of personal morality and the moral context in people’s motivation and perception of violence as an alternative, and we will consider the need to exercise self-control, taking motivation and morality into account, but our focus is on the basic relationships between intoxication, exercising self-control, and violence. We use comparative contemporary samples of 15–16 year-olds from Peterborough, UK, and Malmö, Sweden, to test if differences in adolescents’ general exercise of self-control predict differences in the effects of intoxication on their violence.

## Methods

### Samples

The subjects for this study come from two on-going longitudinal studies, the Peterborough Adolescent and Young Adult Development Study (PADS +) based in Peterborough, UK; and the Malmö Individual and Neighbourhood Development Study (MINDS) based in Malmö, Sweden. For these analyses, data is used only from subjects aged 15–16 years old, a peak age for crime involvement (Wikström et al., 2024).

#### *PADS* + 

PADS + participants were randomly selected from the age cohort of 10–11 year-olds living in Peterborough in 2002 (see Wikström et al., 2012b). Peterborough is a small UK city with a population of 163,000 in 2007 when the data used in this paper was collected (PADS + wave 4) and participants were 15 turning 16. Most (704; 98%) of the 716 participants who take part in wave 1 (72% of the original sampling frame) took part in wave 4. Demographically, participants and their families generally reflect the population of England and Wales (see Wikström et al., 2012b).

##### MINDS

MINDS participants were randomly selected from the age-cohort living in Malmö, Sweden in 2007 and born in 1995. Nearly twice the size of Peterborough with a population (in 2009) of 293,000 inhabitants, Malmö is a commercial centre located in the south of Sweden. Data for this paper was collected in 2010, when participants were also 15 turning 16 (MINDS wave 3); 89% (514)^5^ of an original sample of 576 (58% of the sampling frame) took part.

### Measures

Data for this study was gathered through interviewer-led questionnaires (see Wikström et al., 2012b). Questionnaires were designed to be comparable across studies.

*Violent crime prevalence* was measured by items asking ‘Not counting events when you took money or other things from someone, have you **in the last year** (200X)^6^beaten up or hit someone, for example, punched, kicked or head butted someone (do not count fights with your brother and sister)?’.

*Alcohol use* was measured by items asking ‘Have you in **the last year** (200X) drunk alcohol (cider, beer, wine, spirits) so that you felt drunk?’; ‘**If Yes**, how many times did you drink alcohol so that you felt drunk in the last year (200X)?’. PADS + participants provided a number of times while MINDS participants answered a Likert-scale coded from 1 to 4: ‘once or twice’ (1), ‘a few times (3–5 times)’ (2), ‘many times (6–10 times)’ (3), or ‘very many times (11 or more times)’ (4). For comparability, both measures were converted to a five-point scale including ‘never’ (coded as 0).^7^ In regression analyses we use this alcohol measure in the form of four dummy variables: intoxication 1–2 times, intoxication 3–5 times, intoxication 6–10 times, and intoxication 11 times or more (which we will refer to as ‘frequent intoxication’), with ‘no intoxication’ serving as the reference category.

*(Poor) ability to exercise self-control* is measured using eight items^8^ asking participants the extent to which they agree or disagree with personal statements, such as ‘I often act on the spur of the moment without stopping to think’ – ‘strongly disagree’ (0), ‘mostly disagree’ (1), mostly agree’ (2), strongly agree’ (3). This index is adapted from the Grasmick Scale (Grasmick et al., 1993) to capture participants’ general experience of exercising self-control, as conceptualized by SAT, i.e., demonstrating the capacity for deliberation that underpins the management of moral conflict. Items were summed to create a composite measure, with higher scores representing poorer ability to exercise self-control.^9^ The scale alphas are satisfactory: .76 for PADS + and .68 for MINDS.

We included measures of personal morality and exposure to criminogenic settings to account for the *need* to exercise self-control, as stipulated by SAT’s principle of the conditional relevance of controls.

*(Weak) violence-relevant morality* is measured using two items from a 16 item scale asking how wrong the participant thinks it is for someone his/her age to ‘hit another young person who makes a rude comment’ or ‘use a weapon or force to get money or things from another young person’ – ‘very wrong’ (0), ‘wrong’ (1), ‘a little wrong’ (2), and ‘not wrong at all’ (3) (Wikström et al., 2012b). These items most closely tap into a participants’ personal morality relating specifically to violence.

*Exposure to criminogenic settings* is a composite measure, constructed by summing the z-scores (standardized across all participants) for *time with peers in criminogenic environments* and *peer crime involvement.* This is a complex construction designed to capture both duration and degree of exposure to weak moral contexts through time spent with crime prone peers in settings which are likely to present sources of temptation and provocation (motivators) and in which rules of conduct will be predominantly established and enforced by peers. *Time with peers in criminogenic environments* is measured using three items asking participants how often they and their friends spend time (1) outdoors hanging around together without doing anything in particular, (2) in shopping centres or shopping malls, and (3) in the city centre – ‘never/almost never’ (0); ‘once or twice a week’ (1); ‘most days of the week (3–5 days a week)’ (2); ‘all days, or almost all days, of the week (6–7 days a week)’ (3). Alphas for this scale are relatively low (.51 for PADS + and .53 for MINDS). Its internal consistency could be improved by tapping more specifically into key features of criminogenic environments, such as the lack of supervision (but see Wikstrӧm et al. 2024: 284–306 for a detailed discussion of this exposure measure, in which it is shown that more than 75% of time spent in these environments is unsupervised). However, one key factor is addressed by including a measure of *peer crime involvement* using six items which ask participants if it often happens that their friends are involved in rule-breaking (e.g., skipping school, using illegal substances, getting into fights) – ‘no, never’ (0); ‘yes, sometimes’ (1); ‘yes, often (every month)’ (2); or ‘yes, very often (every week)’ (3). Alphas for this scale were robust: .84 for PADS + and .78 for MINDS.

It has been debated whether self-report measures of peer crime involvement reliably capture participants’ peers’ crime or are a projection of the participants’ own crime (see, e.g., Boman et al. 2014a, 2014b; Walters, 2019; Young et al., 2014). As SAT argues it is a person’s perception of the moral context that influences his/her perception and choice of action alternatives, participants’ perceptions of their peers’ crime involvement, and not their peers’ actual crime involvement (if these differ) is the appropriate measure. Evidence does support the situational influence of participants’ experiences with their peers on their perceptions of their peers’ crime involvement; unique analysis of PADS + space–time budget data shows that participants who report their peers are prone to certain types of crime are exposed to more such crimes when with their peers (Wikstrom et al. 2024: 295–297).

### Analytical Plan

Our analysis proceeds in three steps. First, we examine descriptive statistics and basic correlations across all participants, and then compare these between the British and Swedish samples. Then we perform linear probability models (LPM) with robust standard errors to examine the relationship between alcohol, self-control and violence in the two cities. LPM estimates are comparable between models, preferred to nonlinear models when analyzing interactions, and reflect the percentage point change in the probability of the outcome for a one-unit increase in the predictor (e.g., Gomila, 2021; Mood, 2010; Wooldridge, 2016). Four regression analyses were performed. The first model examined the association between city and violence. The second model examined the association between ability to exercise self-control, intoxication and violence. The third model examined if ability to exercise self-control and intoxication interact in their association with violence. The fourth and final model included measures of violence-relevant morality and criminogenic exposure to account for differences between samples in the need to exercise self-control in relation to violence, and situate the findings in the context of the wider theoretical model we are testing, given this model emphasizes the conditional relevance of self-control and its dependence on the interplay between personal moral rules and the rules of the setting, with relevance for cross-national comparison. To examine whether the association between city and violence significantly differs between models, we performed a cross-model test using the *suest* command in Stata (Clogg et al., 1995; Mize et al., 2019; Weesie, 2000). Analyses were run using SPSS 29.0 and Stata 17.

## Results

Consistent with previous research in the UK and Sweden, we find that adolescents from Peterborough were significantly more likely to report violence than adolescents from Malmö (see Table 1).^10^ Adolescents from Peterborough were also much more likely to report having been drunk in the previous year and to report frequent intoxication (Table 1).

**Table 1:** Descriptive statistics

	Total		PADS +		MINDS			
	M/%	SD	M/%	SD	M/%	SD	t–test; χ^2^	
*Violent crime*								
Prevalence (%)	16.30	–	22.9	–	7.6	–	48.53	^***^
*Explanatory variables*								
Alcohol prevalence (%)	59.8	–	73.7	–	40.7	–	134.3	^***^
Drunk 11 + times (%)	25.1	–	35.5	–	10.9	–	95.4	^***^
Self–control	10.43	4.24	10.59	4.32	10.22	4.12	1.47	
Violence–relevant morality	1.38	1.14	1.57	1.16	1.12	1.07	7.74	^***^
Exposure	1.19	1.64	.30	1.70	–0.43	1.45	7.74	^***^

^*^p < .05, ^**^p < .01, ^***^p < .001

Despite these substantive behavioural differences, no significant difference was found in the samples’ ability to exercise self-control, implying that their general levels of executive functioning were similar, as might be expected from representative populations. However, adolescents from Peterborough did report weaker violence-relevant morality and greater exposure to criminogenic settings (Table 1).

Across all participants, violence correlated positively with the ability to exercise self-control, intoxication frequency, violence-relevant morality, and criminogenic exposure (*p* < .01) (Table 2).^11^ The ability to exercise self-control correlated with intoxication frequency, indicating that those with less ability to exercise self-control tended to get drunk more often; and with violence-relevant morality and criminogenic exposure, suggesting that these factors may be concentrated in a particular group of people who share specific time use patterns. Frequent intoxication also correlated positively with violence-relevant morality and criminogenic exposure, consistent with the idea that selection effects may influence the kinds of people who drink regularly and heavily and where they spend their time.

**Table 2:** Bivariate correlations (Spearman’s rho) for all participants and in parentheses by study (PADS + /MINDS)

		1	2	3	4	5	6	7	8	9	10
1	Violent crime prevalence	1									
2	Intoxication frequency	.31^**^ (.29^**^/.21^**^)	1								
3	Intoxication prevalence	.23^**^ (.19^**^/.18^**^)	.90^**^ (.79^**^/.97^**^)	1							
4	Intoxication 1–2 times	–.03 (–.05/.02)	–.03 (–.23^**^/.24^**^)	.30^**^ (.21^**^/.45^**^)	1						
5	Intoxication 3–5 times	–.04 (–.10^**^/.04)	.14^**^ (–.07/.36^**^)	.34^**^ (.26^**^/.42^**^)	–.14^**^ (–.16^**^/–.12^**^)	1					
6	Intoxication 6–10 times	–.01 (–.02/.02)	.25^**^ (.11^**^/.40^**^)	.27^**^ (.21^**^/.36^**^)	–.11^**^ (–.12^**^/–.10^*^)	–.13^**^ (–.15^**^/–.09^*^)	1				
7	Intoxication 11 + times	.32^**^ (.30^**^/.21^**^)	.78^**^ (.86^**^/.60^**^)	.48^**^ (.44^**^/.43^**^)	–.20^**^ (–.26^**^/–.12^**^)	–.22^**^ (–.33^**^/–.11^**^)	–.18^**^ (–.26^**^/–.09^*^)	1			
8	Poor self–control	.28^**^ (.30^**^/.27^**^)	.33^**^ (.31^**^/.39^**^)	.29^**^ (.21^**^/.38^**^)	–.03 (–.13^**^/.09^*^)	.07^*^ (–.01/.17^**^)	.08^**^ (.01/.17^**^)	.25^**^ (.28^**^/.21^**^)	1		
9	Weak morality	.28^**^ (.26^**^/.23^**^)	.30^**^ (.26^**^/.24^**^)	.25^**^ (.17^**^/.23^**^)	–.02 (–.08^*^/.06)	.03 (–.05/.10^*^)	.03 (.00/.05)	.26^**^ (.25^**^/.17^**^)	.29^**^ (.29^**^/.27^**^)	1	
10	Criminogenic exposure	.34^**^ (.32^**^/.27^**^)	.46^**^ (.42^**^/.44^**^)	.41^**^ (.31^**^/.44^**^)	.01 (–.12^*^/.17^**^)	.04 (–.06/.13^**^)	.09^**^ (.04/.16^**^)	.37^**^ (.38^**^/.25^**^)	.42^**^ (.38^**^/.25^**^)	.31^**^ (.35^**^/.20^**^)	1

^*^p < .05, ^**^p < .01

These correlation patterns held across both samples (Table 2, in parentheses) except that for adolescents in Peterborough a low intoxication frequency (1–2 times in the past year) correlated *negatively* with a poor ability to exercise self-control, weak violence-relevant morality, and high criminogenic exposure, while only frequent intoxication (11 + times) correlated positively, suggesting, as does the prevalence of alcohol use amongst Peterborough’s teens (74%), that only some adolescents only get drunk less than three times a year, and these tend to have a particularly good ability to exercise self-control, strong morality, and low criminogenic exposure. By contrast, for adolescents in Malmö any intoxication correlated *positively* with a poor ability to exercise self-control, weak morality and criminogenic exposure.

Table 3 presents four LPM regression models. Model 1 shows a small but significant proportion (4.1%) of the variance in violence in the total sample can be predicted by the city. However, the association between city and violence declines significantly when the ability to exercise self-control and intoxication frequency are introduced in model 2. A poor ability to exercise self-control and frequent alcohol use independently predict a higher likelihood of .

**Table 3:** The association between violence and self-control, intoxication, morality and exposure

	Model 1	Model 2	Model 3	Model 4
	b	b	b	b
Study (1 = MINDS)	–.149^***^	–.090^***^	–.093^***^	–.042
Self–control		.019^***^	.009^**^	.004
Intoxication 1–2 times^a^		.041	.056	.077
Intoxication 3–5 times^a^		.000	.013	.007
Intoxication 6–10 times^a^		.033	.049	.049
Intoxication 11 + times^a^		.205^***^	.190^***^	.170^***^
Self–control × Intoxication 1–2 times			.012	.013
Self–control × Intoxication 3–5 times			.014	.013
Self–control × Intoxication 6–10 times			.009	.006
Self–control × Intoxication 11 + times			.026^***^	.024^***^
Morality				.036^**^
Exposure				.034^***^
R^2^ × 100	4.0%	16.4%	17.7%	21.0%

Linear probability model using robust standard errors. Model 4 controls for the interaction between study × self-control and study × intoxication (not shown)

^a^Reference category = Never been drunk last year

^*^p < .05; ^**^p < .01; ^***^p < .001

Model 3 explores the interaction between participants’ ability to exercise self-control and intoxication frequency in their association with violence. Only the interaction with frequent intoxication is significant (*b* = .026, *p* < .001). The positive interaction term suggests that the relationship between frequent intoxication and violence depends on one’s ability to exercise self-control; the poorer one’s ability, the greater the relationship between intoxication and one’s violence. The fact that no effects were detected for lower rates of intoxication may be a consequence of these participants not having enough opportunities for alcohol to interact with their ability to exercise self-control in circumstances which led to violence, that they were not exposed to criminogenic settings when intoxicated, or that frequently intoxicated adolescents represent a select group, for example, with particularly weak morality or high exposure to criminogenic settings, who have a greater *need* to exercise self-control, and therefore it matters more to their violent outcomes.

To address this possibility, we looked at whether the relationship between adolescents’ alcohol use, ability to exercise self-control, and violence was attenuated once their personal morality and criminogenic exposure were taken into account (Model 4).^12^ The results show that no significant association remains between city and violence once morality and exposure are included, indicating there is no direct association between city and violence. In addition, frequent intoxication continues to be associated with violence for those with a poorer ability to exercise self-control.

## Discussion and Conclusions

In this paper we have examined the relationships between alcohol use, the ability to exercise self-control, and violence in adolescents in the UK and Sweden. Based on the propositions of SAT, we hypothesized that alcohol use would be associated with violence in the same way in both samples: by reducing adolescents’ ability to exercise self-control and choose alternative responses when they perceived violence as a possible action alternative. We also hypothesized that the association between alcohol use and violence would depend on a person’s ability to exercise self-control: for those with a poorer ability to exercise self-control generally, we expected further reductions as a consequence of intoxication would increase their likelihood of choosing violence as an alternative. Both hypotheses were supported; frequent intoxication and a poorer ability to exercise self-control were significantly associated with a greater likelihood of violence, and the relationship between intoxication and violence was stronger the weaker an adolescent’s general ability to exercise self-control.

We also drew on SAT’s principle of the conditional relevance of controls (e.g., Wikstrom & Treiber 2007), one of several facets of SAT’s explanation of the role of self-control in crime involvement that distinguishes it from GTC’s (Gottfredson and Hirschi 1990), and included measures of personal morality and criminogenic exposure to account for differences in adolescents’ *need* to exercise self-control. We found that once we accounted for the ability to exercise self-control, including the impact of intoxication, and the need to exercise self-control, via personal morality and criminogenic exposure, participants’ city was no longer relevant for predicting their violence. This suggests that British and Swedish youths differ in their *need* to exercise self-control to choose non-violent alternatives, as a consequence of differences in their propensities to see violence as an alternative and exposure to settings that activate those propensities. Once these differences are taken into account, there are no differences by country in the relationship between adolescents’ ability to exercise self-control, frequency of intoxication, and choice of violence as an alternative. This is consistent with SAT’s proposition that its perception–choice process is universal, while the inputs to that process will vary by population and context (Wikström, 2010).

The significant differences observed in alcohol use and violence between Swedish and British youths cannot be explained by the lack of significant differences in their ability to exercise self-control; therefore, it does not appear to be the case that alcohol use and violence can be seen as direct outcomes or manifestations of differences in these samples’ ability to exercise self-control. We have posited a more nuanced explanation for their interrelationship, delineating different casual factors, their distinct causal roles, and the causal processes that link them together, and empirically demonstrated interaction effects that support this less parsimonious but more detailed model.

Testing situational interactions, particularly the convergent interactions posited in SAT, creates significant challenges that often cannot be addressed using traditional data or analytical methods (Hardie, 2020). To meet these challenges, ongoing dialogue is needed between theory, research design, data collection methods, and analytical techniques. In this paper we present a basic preliminary cross-comparative examination of one specific component of a much larger and more complex model.

Importantly, regression methods such as those presented in this paper cannot directly tap into situational processes. Neither the data presented nor the models employed can specify whether or not the violence being analyzed took place when a person with a poor ability to exercise self-control was intoxicated. To test this directly requires situational data that places people – and their actions – in specific settings, such as that collected using the space–time budget method (Wikström et al., 2012a, 2012b). PADS + and MINDS both collected space–time budget data, making this a possible direction for future research. However, due to its rarity, the number of violent acts captured by the space–time budget method is small. Thus regression methods such as those presented in this paper provide valuable insights into the person–environment interaction. Cross-sectional analyses such as those presented in this paper cannot clarify the sources of poor ability to exercise self-control and high rates of alcohol use, or how these may influence one another developmentally. This is another direction for future research to which PADS + and MINDS longitudinal data may contribute.

More research is needed to explore these relationships, situationally and developmentally, before strong conclusions can be drawn and recommendations made for where and how to intervene. However, a broad implication of our findings is that the same kinds of intervention may be effective for both British and Swedish youths. Our findings corroborate well-known recommendations to minimize alcohol use during adolescence, prevent exposure to settings conducive to violence, and foster the ability to exercise self-control as well as attitudes that discourage violence. They suggest that improving adolescents’ ability to exercise self-control and reducing their alcohol use may provide a second line of defense against violence by those who need to exercise self-control because their first line of defense fails – i.e., their personal morals or the settings they take part in fail to discourage violence.

## Data Availability

Data sharing is not applicable to this article as no new data were created or analysed in this study.

## Footnotes

1 *Situation* refers to the interaction between a person and a setting (his/her immediate environment), and therefore situational factors to characteristics of the *interaction* (Wikström & Kroneberg, 2022).

2 Notably, Skåne, the county in which Malmö is located, has one of the highest rates of victimization across Swedish counties according to the Swedish Crime Survey 2012.

3 The ability to be deterred is related to the ability to exercise self-control as it relies on the same neurocognitive substrates. Thus the effects of intoxication might be seen across the ability to be controlled more generally.

4 The failure of some studies (for reviews see Hardie & Rose, 2025; Pauwels et al., 2018) to find this interaction may be attributable to their use of self-control measures which are confounded with personal morality; and/or because they use selective samples.

5 Two outliers were excluded when close inspection suggested their pattern of responses was unreliable.

6 In MINDS participants were asked ‘in the last school year (school year X)’.

7 Analyses were run three ways: using (1) this raw scale, (2) mean values (e.g., 0, 1, 4, 8, 11), and (3) lowest values (0, 1, 3, 6, 11) per category. Although models were slightly improved using the latter two values, differences were minimal.

8 ‘When I am really angry, other people better stay away from me’; ‘I often act on the spur of the moment without stopping to think’; ‘I sometimes find it exciting to do things that may be dangerous’; ‘I do not devote much thought and effort preparing for the future’; ‘Sometimes I will take a risk just for the fun of it’; ‘I often try to avoid things that I know will be difficult’; ‘I never think about what will happen to me in the future’; ‘I lose my temper pretty easily’.

9 Missing items were minimal; if a participant was missing no more than two items, mean values were imputed from existing items.

10 At rates extremely similar to those reported by Wikström and Svensson (2008) comparing the Peterborough Youth Study and the Eskilstuna Youth Study.

11 Multicollinearity was not a problem in any of our analysis; the highest Variance Inflation Factor Score was 3.40 for the total sample, 3.56 for Peterborough, and 1.90 for Malmö.

12 We also ran models exploring the interaction between morality, self-control, and exposure. This did not significantly change the findings.
